# Mixed Acinar-Neuroendocrine Carcinoma of the Pancreas with Neuroendocrine Predominance

**DOI:** 10.1155/2013/705092

**Published:** 2013-11-17

**Authors:** Onyekachi Henry Ogbonna, Marie Carmel Garcon, Kostas N. Syrigos, Muhammad Wasif Saif

**Affiliations:** ^1^Section of GI Cancers and Experimental Therapeutics, Tufts University School of Medicine, Boston, MA 02111, USA; ^2^Columbia University Medical Center, New York, NY 10032, USA; ^3^Oncology Unit, Third Department of Medicine, Athens University, School of Medicine, Sotiria General Hospital, 115 27 Athens, Greece

## Abstract

*Background*. Pancreatic tumors are rare and could arise from either the exocrine (ductal and acinar cells) or the endocrine (neuroendocrine cells) components of the pancreas. In some instances, the occurrence of pancreatic tumors comprising both acinar cells and neuroendocrine cells, with neuroendocrine cells making up more than 30% of the tumor, has been identified. This unique entity has been referred to as mixed acinar-neuroendocrine carcinoma (MANEC). Only about 20 such cases have been reported in the literature. *Case Report*. We report an interesting case of MANEC with neuroendocrine cell predominance in a woman presenting with epigastric pain secondary to a pancreatic mass with acinar and endocrine differentiation. She underwent surgical resection of the tumor and was offered adjuvant treatment chemotherapy with carboplatin, etoposide, and radiotherapy for positive tumor resection margins. *Conclusions*. Given the paucity of the cases of MANEC, continuous reporting of these cases when identified should be encouraged to aid oncologists in understanding the disease and help establish standardized management.

## 1. Introduction

Pancreatic tumors are rare and constitute less than 1% of all neoplasms. Adenocarcinomas represent more than 75% of these tumors, with neuroendocrine carcinomas accounting for 7% and acinar cell carcinomas accounting for 1% [1, 2]. Mixed endocrine-exocrine tumors of the pancreas have also been described but are very rare, with less than 20 cases reported in the English literature, and they show a predominantly acinar pattern. We present a case of a 57-year-old woman presenting with a mixed acinar-neuroendocrine carcinoma of the pancreas with a predominant neuroendocrine component.

## 2. Case Report

The patient is a 57-year-old woman who presented with nagging epigastric pain, worse at night, and radiating to the back. She denied fevers or chills, malaise, fatigue, or weight loss. She denied family history of pancreatic cancer but reported a history of ovarian cancer in her mother (at age 68) and colon cancer in her maternal grandfather (at age 72). Abdominal exam was unremarkable. She eventually underwent abdominal Magnetic Resonance Imaging which showed a 2.5 cm mass in the body of her pancreas. Cancer antigen 19-9 (CA19-9), carcinoembryonic antigen (CEA), alpha-fetoprotein (AFP), and serotonin levels were normal. Chromogranin A level was 122 U/L (normal range 0–95 U/L) and pancreatic polypeptide level was 767 U/L (normal range 0–435 U/L). Endoscopic ultrasound-guided fine needle aspiration of the mass was performed and was positive for pancreatic carcinoma. She then underwent distal pancreatectomy and splenectomy. Pathological examination of the surgical specimen revealed a poorly circumscribed, mostly solid neoplasm with mixed acinar-neuroendocrine morphology, predominantly neuroendocrine. The tumor was high grade (grade 3), with focal necrosis, measuring 2.5 cm in the greatest dimension, extending in peripancreatic adipose tissue. It extended beyond the pancreas but without the involvement of the celiac axis or the superior mesenteric artery. No regional lymph node metastasis was identified. Immunohistochemical staining was positive for cytokeratin, synaptophysin, and chromogranin and weakly positive for chymotrypsin. Trypsin staining was noncontributory. Areas of the tumor with acinar architecture were periodic acid-Schiff positive (PAS+); Ki-67 had 40–45% positivity. She also underwent an octreotide scan after surgery which showed no evidence of uptake. Given the fact that the retroperitoneal margin of her surgical resection was positive for cancer, the plan was to start her on adjuvant therapy involving 6 cycles of etoposide/carboplatin and radiation. She delayed the treatment for a trip but did not come back to the clinic subsequently because she was not ready to accept the diagnosis, the prognosis, or the treatment plan.

## 3. Discussion

The pancreas is made up of exocrine and endocrine gland components, with the exocrine part comprising ductal and acinar cells and the endocrine part comprising endocrine cells. Ductal adenocarcinomas account for the vast majority of pancreatic cancers—more than 75%—with neuroendocrine carcinomas accounting for 7% and acinar cell carcinomas accounting for about 1% of known pancreatic cancers [1, 2], although the pancreas is composed predominantly of acinar cells [2]. Acinar cell carcinomas (ACC) of the pancreas have been known to express neuroendocrine markers as well in up to third of the cases, but these are generally limited to only a few neuroendocrine cells [1]. Occasionally, however, the neuroendocrine cells may comprise over 30% of the tumor mass in which case the tumor is referred to as a different entity—mixed acinar-neuroendocrine carcinoma (MANEC) [1, 3, 4].

Mixed acinar-neuroendocrine carcinomas are incredibly rare with only about 20 or so cases in the published literature [1]. They tend to have a predominantly acinar pattern. In our case, our patient presents with a predominantly neuroendocrine component, which is even more uncommon and makes the case more interesting. MANECs are large tumors, most commonly located at the head of the pancreas (60% of the cases) [1, 4], and are often diagnosed in middle-aged individuals (mean age of 58) [1]. Unlike ACC, which is more common in men, MANEC appears to be more common in women [4–6], although this may be incidental, limited by the small number of MANEC cases that have been reported. Patients present with vague abdominal pain and weight loss and rarely present with an endocrine tumor [1, 7, 8]. Jaundice is relatively uncommon since it is a well-circumscribed tumor [4].

The diagnosis is made by pathological analysis of the tumor morphology and immunohistochemical staining. Tumor cells for ACC stain positively for trypsin, chymotrypsin, and lipase and are periodic acid-Schiff positive (PAS+). Tumor cells for pancreatic neuroendocrine tumors stain positively for chromogranin and synaptophysin [4]. In our case, our patient stained positively for PAS, chymotrypsin, Chromogranin, and synaptophysin, indicative of tumor differentiation towards both acinar and neuroendocrine carcinomas.

Due to the small number of cases of MANEC reported, no standardized management protocol has been established. However, it is generally agreed that surgery is the first line of treatment for all cases with resectable tumor [1]. There have also been reports of patients benefiting from surgical tumor debulking and local and systemic antiproliferative therapy [1]. In the treatment of acinar cell carcinoma, the results of adjuvant chemotherapy and radiotherapy have been disappointing [9]. It has been suggested that the presence of a neuroendocrine component in ACC may be related to a more favorable outcome [1]. The prognosis of patients with MANEC is generally poor as with other pancreatic cancers; the mean survival of MANEC patients after surgical resection of the primary tumor is 10.5 months although there is one case of a patient with MANEC initially presenting with Zollinger Ellison syndrome who lived for 24 years after surgical resection and died of disseminated carcinomatosis [1, 7]. Given her high-grade tumor with high number of mitoses and high Ki-67 index on histology, our patient has a very poor prognosis. The presence of cancer in her retroperitoneal surgical margin is also a poor prognostic indicator.

## 4. Conclusions

MANECs are very rare tumors of the pancreas for which surgical resection remains the only established standard or treatment. Continued identification and reporting of these cases should be encouraged with the goal of understanding the disease better and standardizing optimal therapy.
